# Pan-genome Analysis of *WOX* Gene Family and Function Exploration of *CsWOX9* in Cucumber

**DOI:** 10.3390/ijms242417568

**Published:** 2023-12-17

**Authors:** Shuai Yin, Lili Zhao, Jiaqi Liu, Yanjie Sun, Bohong Li, Lina Wang, Zhonghai Ren, Chunhua Chen

**Affiliations:** 1 College of Horticulture Science and Engineering, Shandong Agricultural University, Tai’an 271018, China; yinshuaicau@163.com (S.Y.); 2020010080@sdau.edu.cn (L.Z.); 17860295685@163.com (J.L.); syj15550853801@163.com (Y.S.); l15531303812@163.com (B.L.); lnwang@sdau.edu.cn (L.W.); zhren@sdau.edu.cn (Z.R.); 2 Department of Vegetable Science, College of Horticulture, China Agricultural University, Beijing 100193, China

**Keywords:** cucumber, WOX, Tubercule formation, fruit-specific

## Abstract

Cucumber is an economically important vegetable crop, and the warts (composed of spines and Tubercules) of cucumber fruit are an important quality trait that influences its commercial value. WOX transcription factors are known to have pivotal roles in regulating various aspects of plant growth and development, but their studies in cucumber are limited. Here, genome-wide identification of cucumber *WOX* genes was performed using the pan-genome analysis of 12 cucumber varieties. Our findings revealed diverse *CsWOX* genes in different cucumber varieties, with variations observed in protein sequences and lengths, gene structure, and conserved protein domains, possibly resulting from the divergent evolution of *CsWOX* genes as they adapt to diverse cultivation and environmental conditions. Expression profiles of the *CsWOX* genes demonstrated that *CsWOX9* was significantly expressed in unexpanded ovaries, especially in the epidermis. Additionally, analysis of the *CsWOX9* promoter revealed two binding sites for the C_2_H_2_ zinc finger protein. We successfully executed a yeast one-hybrid assay (Y1H) and a dual-luciferase (LUC) transaction assay to demonstrate that *CsWOX9* can be transcriptionally activated by the C_2_H_2_ zinc finger protein Tu, which is crucial for fruit Tubercule formation in cucumber. Overall, our results indicated that *CsWOX9* is a key component of the molecular network that regulates wart formation in cucumber fruits, and provide further insight into the function of *CsWOX* genes in cucumber.

## 1. Introduction

The *WUSCHEL-related homeobox* (*WOX*) gene family belongs to the plant-specific subgroup of the homeodomain (HD) superfamily [1,2]. *WOX* genes have been identified in various plant species, such as *Arabidopsis*, apple, maize, rice, and soybean [1,3,4,5,6]. WOX transcription factors (TFs) possess a typical HD that contains 60–66 amino acids with a helix–loop–helix–Turn–helix structure [2,5,7]. Based on the type of HD and the presence or absence of the WUSCHEL (WUS)-box, WOX TFs can be classified into three phylogenetically distinct clades: the WUS clade, which contains a WUS-box and a representative motif FYWFQNH in the HD; the intermediate clade, which contains a representative motif FYWFQNR in the HD and lacks a WUS-box; and the ancient clade, which contains a representative motif YNWFQNR in the HD and also lacks a WUS-box [5,8].

WOX TFs play crucial roles in regulating various aspects of plant growth and development [9,10,11]. Many members of the WOX family are known to regulate stem cell formation and maintenance in seed plants. WUS is necessary for maintaining the homeostasis of the stem cell population in shoot apical meristems [12,13]. Similarly, WOX5, which is specifically expressed in the root apical meristems (RAMs) quiescent center, and WOX4 are also involved in stem cell maintenance [14,15,16]. In *Medicago truncatula* and pea (*Pisum sativum*), the *WOX5* gene was expressed during nodule organogenesis and might control cell proliferation and differentiation in nodule meristems [17]. Additionally, WOXs are involved in plant development processes such as root, flower, and fruit development. For instance, rice OsWOX11 recruits the histone acetyltransferase complex ADA2-GCN5 to regulate crown root cell proliferation [18]. In *Arabidopsis*, *AtWOX7* plays an important role in integrating nutrient status with the lateral root formation program [19]. In rice, *OsWOX4* controls primary root elongation by influencing the size of root meristem as well as the cell length in the mature zone in the root tip via the auxin signaling pathway [20]. Furthermore, *PtoWOX5a* and *PtoWUSa* from poplar have been identified as critical regulators of root development [21,22]. Tomato *UF*, which encodes the SlWOX1 protein, is involved in controlling flower and leaf lateral growth [23]. Moreover, *WOX* genes such as *OsWOX13* from rice, *AtWOX13* and *AtWOX14* from *Arabidopsis*, and *EVERGREEN* (*EVG*) from *Petunia*, are known to play important roles in flower development. Additionally, the *PRETTY FEW SEEDS2* (*PFS2*/*WOX6*) gene is primarily expressed throughout developing ovules and regulates ovule development [24,25]. *CsWOX9* is mainly expressed in developing fruit in cucumber, and its overexpression in *Arabidopsis* leads to shorter siliques [26].

Cucumber (*Cucumis sativus* L.) is one of the most economically important vegetable crops that produces edible tender fruits. The visual appearance of cucumber fruit is a crucial factor in determining its economic value. This includes characteristics such as peel color, fruit size, and the presence or absence of warts, which are composed of spines and Tubercules. Warts are a distinctive trait that sets cucumber apart from other fruits in the Cucurbitaceae family. Moreover, warts are also an important quality trait that impacts the market value of cucumber [27,28]. Consequently, studying the genes involved in wart formation is important for improving breeding to acquire desirable agronomic traits and enhance economic value of cucumber production. Given the WOX proteins play an important role in regulating plant growth and development, there is growing interest in identifying functional *CsWOX* genes that regulate wart formation. Based on the cucumber 9930 genome v2.0 or v3.0, 11 *CsWOX* genes have been identified, and some of these genes have shown organ-specific expression patterns [26,29].

Recently, there has seen a surge in the study of functional genomics, and the high degree of genomic variation observed, leading to the realization that a single reference genome does not reflect the diversity within a species [30,31,32,33]. Thus, the concept of the pan-genome, originally proposed in bacteria, has been expanded [34]. Pan-genomic analysis is now widely used in plant, fungal, and animal genomics to assess genetic diversity within species, as well as to explore cross-species gene exchange and domestication and improvement processes [31,32,33,34]. 

In cucumber, a graph-based pan-genome was constructed by analyzing 12 chromosome-scale genome assemblies [35]. Consequently, a new genome-wide identification of cucumber *WOX* genes was performed using the pan-genome of these 12 cucumber varieties. Comprehensive analyses, including of the protein sequences and lengths, gene structure, and conserved protein domains, were further performed. Additionally, the expression levels of *CsWOX* genes during cucumber fruit development were investigated. We found that *CsWOX9* is mainly expressed in the epidermis of cucumber ovaries. Further studies revealed that the Tuberculate fruit gene *Tu* directly activates *CsWOX9* by binding to its promoter.

## 2. Results

### 2.1. Identification of WOX Genes Based on Pan-genome of Cucumber

**Figure 1:**
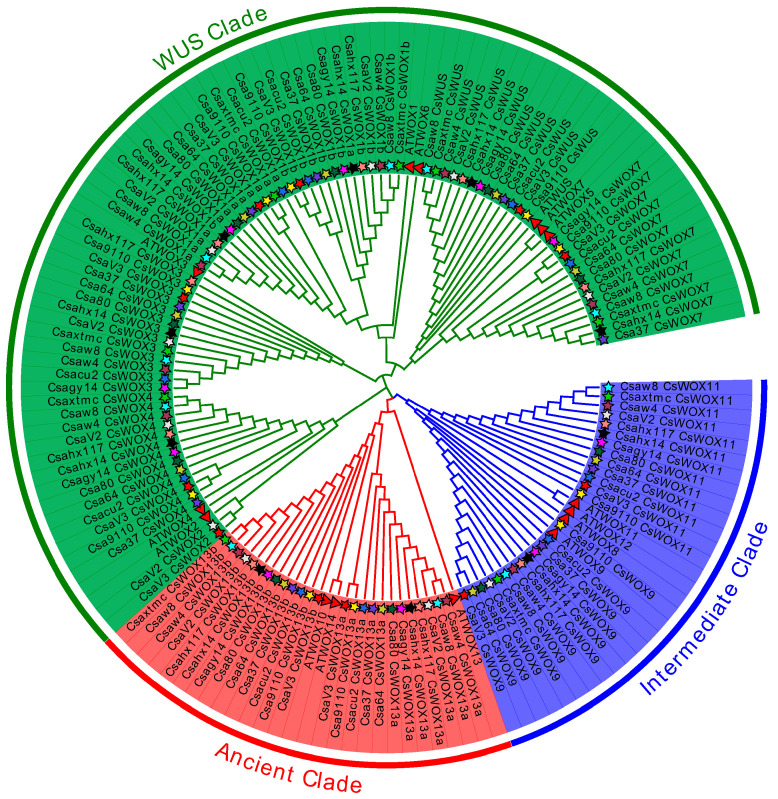
The phylogenetic tree of 131 CsWOX proteins from 12 cucumber varieties and 15 AtWOX proteins, indicating that CsWOXs could be divided into 3 clades. The full lengths of all WOX proteins were used for phylogenetic analysis by MEGA7.0 software with the neighbor-joining (NJ) method. Three clades are represented by different colors. The star and triangle represent WOXs from cucumber and *Arabidopsis*, respectively. The stars with different colors represent different cucumber varieties.

To investigate the potential function of the *WOX* genes in cucumber, we first identified the number of *CsWOX* genes in 12 different cucumber varieties. Consistent with previous studies [26,29], we identified 11 putative *WOX* genes in cucumber 9930 (genome v2.0 and v3.0) (Table 1). There were 10 *CsWOX* genes (not including *CsWOX2*) obtained and further confirmed in Cu2, Cuc37, Cuc64, Cuc80, W4, W8, Hx14, Hx117, Gy14, and 9910gt (Table 1). In cucumber variety XTMC, only nine *CsWOX* genes (excluding *CsWOX2* and *CsWOX13a*) were identified (Table 1). This discrepancy might arise from distinct evolutionary selection processes within various cultivation environments. Additionally, a phylogenetic tree was constructed, showing that these *CsWOX* genes, including 131 *CsWOX* genes from 12 cucumber varieties and 15 *AtWOX* genes, were divided into 3 classes (Figure 1). This result is consistent with previous studies in *Arabidopsis* and cucumber [1,25,28]. 

In order to investigate whether the *CsWOX* genes differed among different cucumber varieties, the predicted lengths of CsWOX proteins were analyzed. As shown in Table 2, the *CsWOX2* gene identified only in the cucumber variety 9930 was consistent in protein sequence and length in genome v2.0 and v3.0. However, other *CsWOX* genes displayed diversity in protein length across these 12 cucumber varieties. The longer length of CsWOX3 and CsWOX11 in W8, as well as CsWOX4 in Cuc64, compared to these CsWOXs in the other varieties, may be a result of termination codon delay or fragment insertion, whereas the shorter length of the CsWOX4 in W8, CsWOX7 in XTMC, CsWOX9 in 9910gt, and CsWOX13a in 9930 (genome v3.0) might be due to termination codon advancement or fragment deletions.

### 2.2. Gene Structure and Motif Composition of CsWOXs

**Figure 2:**
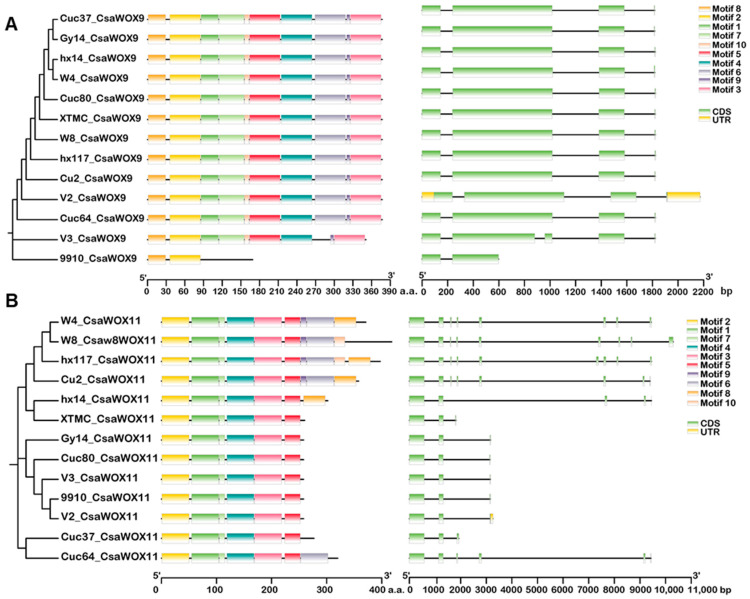
Comparison of the conserved motifs and gene structures of intermediate clade genes *CsWOX9* (**A**) and *CsWOX11* (**B**) in 12 cucumber varieties. Left panel: the phylogenetic tree was constructed with the full length of WOX proteins using MEGA 7.0. Middle panel: the conserved motifs for CsWOX proteins. The motifs 1–10 are highlighted with different colors. Right panel: exon-intron structure of *CsWOX* genes. Black lines and light green boxes represent introns and exons, respectively. CDS, coding sequence; UTR, untranslated region; aa, amino acid; V2, cucumber 9930 genome v2.0; V3, cucumber 9930 genome v3.0; 9910, cucumber 9910gt.

Gene structural diversity can reflect the evolution of multigene families [36]. Therefore, we analyzed the exon-intron organization of the *CsWOX* genes, which vary in protein length in at least three different cucumber varieties. These include the ancient clade gene *CsWOX13b*, intermediate clade genes *CsWOX9* and *CsWOX11*, and WUS clade genes *CsWOX1a*, *CsWOX4*, *CsWOX7*, and *CsWUS*. In the ancient and WUS clades, we observed that the *CsWOXs* had one to four exons, and most members in different cucumber lines shared the same or similar intron/exon arrangements (Figures S1 and S2). For example, *CsWOX13b* contained two introns in all cucumber lines, with the exception of the 9930 genome v3.0, which had only one intron (Figure S1). *CsWOX7* had no introns in the cucumber lines XTMC and 9930 (genome v2.0), but other varieties contained one intron (Figure S2). As shown in Figure 2A, *CsWOX9* of the intermediate clade had three introns in most cucumber varieties. The number of introns in *CsWOX11* of the intermediate clade gene varied across the 12 different cucumber varieties, ranging from 2 to 8. Notably, all Indian cucumber lines, including Cuc64, W4, W8, Hx14, and Hx117, contained at least five introns. 

To better understand the conservation and diversification of *CsWOX genes* across different varieties, the protein sequences of *CsWOX* genes, selected for structure analysis, were used for MEME motif analysis to identify conserved motifs. As expected, CsWOXs with similar or identical protein lengths exhibited comparable motif compositions (Figure 2, Figures S1 and S2). For instance, in the WUS clade gene *CsWOX4,* the protein length ranged from 226 to 228 amino acids (a.a.), and there were no differences in exon-intron organization and conserved motifs (Figure S1). Regarding the ancient clade gene *CsWOX13b*, the protein length in cucumber varieties Cuc37, Cuc80, W4, W8, Hx14, 9910gt, and Hx117 (encoding the protein length of 317 a.a.) increased compared to that in other varieties (encoding the protein length of 269 a.a.), while the gene structure remained unchanged. However, the number of conserved motifs increased. Motif 5 and motif 9 were found to be specific to *CsWOX13b* encoding the longer protein length of 317 a.a. (Figure S2). The protein length of the intermediate clade gene *CsWOX9* was shorter in 9910gt and 9930 (genome v3.0). Furthermore, the gene structure underwent changes and some conserved motifs were absent (Figure 2). Notably, there were variations in protein length, gene structure, and conserved motifs of *CsWOX7*, *CsWOX9*, and *CsWOX13b* between the 9930 genome v2.0 and v3.0 (Figure 2, Figures S1 and S2), potentially due to the incomplete and low quality of genome v2.0.

### 2.3. Multiple Sequence Alignment of CsWOXs

**Figure 3:**
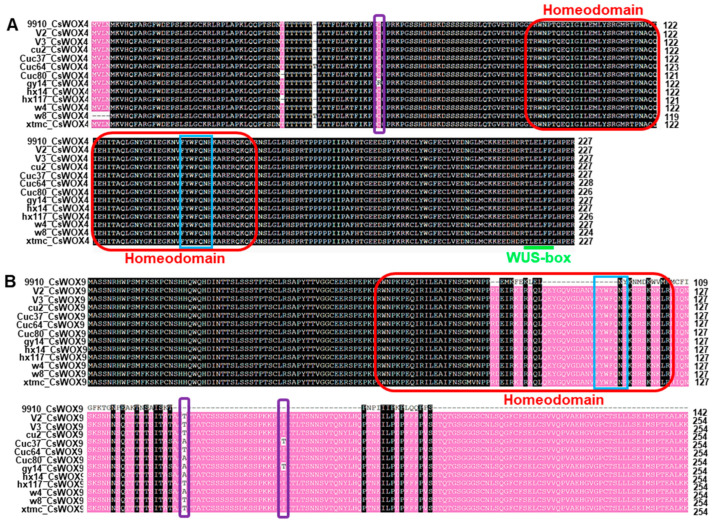
Alignment of multiple amino acid sequences of CsWOX4 and CsWOX9 from 12 cucumber varieties. (**A**) The sequence alignment of CsWOX4. (**B**) The sequence alignment of CsWOX9. The red box indicates homeodomain and the blue box represents conserved motifs in homeodomain. The purple box indicates amino acid polymorphism. The WUS-box is shown by a green line.

In order to investigate whether there were differences in the sequence and conservation of the domain for the same CsWOX member in different cucumber lines, we selected the WUS clade member CsWOX4 and the intermediate clade member CsWOX9 as representatives for analysis. The alignment results showed that all members of CsWOX4 and CsWOX9 contained a conserved HD that carried a helix–loop–helix–Turn–helix structure, except for CsWOX9 in cucumber variety 9910gt (Figure 3A,B). We discovered that the representative motifs FYWFQNH and WUS-box were present in all members of CsWOX4 (Figure 3A). However, the intermediate clade gene *CsWOX9* did not have a WUS-box, and the representative motif of HD also had a single amino acid substitution: H for R (FYWFQNR), except for *CsWOX9* in the cucumber variety 9910gt, which had a deletion of fragments, resulting in an incomplete HD (Figure 3B). Furthermore, we found that the sequences of CsWOX4 and CsWOX9, which had the same or similar protein lengths in different cucumber varieties, were not identical (Figure 3A,B). These variations possibly stem from the divergent evolution of *CsWOX* genes as they adapt to diverse cultivation and environmental conditions.

### 2.4. Identification of Fruit-Specific Expressed Gene CsWOX9

**Figure 4:**
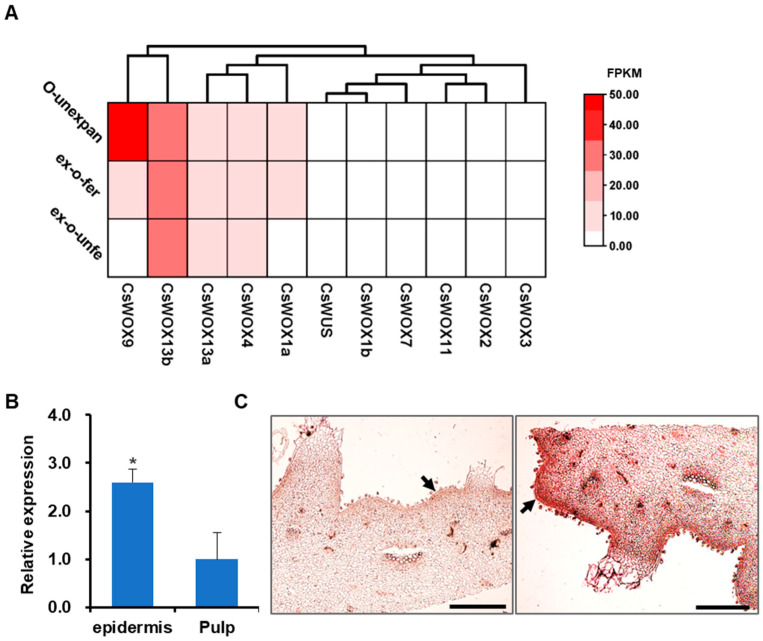
Expression pattern of *CsWOX* genes in cucumber. (**A**) The transcriptional levels of *CsWOX* genes in different developmental stages were investigated based on a public transcriptome data. The FPKM value of each gene was used to make the heat map. The color scale shows the increasing expression levels from white to red. O-unexpan, unexpanded ovary; ex-o-fer, expanded fertilized ovary; ex-o-unfer, expanded unfertilized ovary. (**B**) qRT-PCR analysis of *CsWOX9* expression in epidermis and pulp of the cucumber ovary at 7 DBA. The cucumber *UBI* gene was used as an internal standard. (*) indicates significant differences at *p* < 0.05 (Student’s *t*-test). (**C**) mRNA in situ hybridization of *CsWOX9* in cucumber line 9930 ovaries at 7 DBA. Left, negative control using the sense probe. Right, strong signal was detected in the epidermis using antisense gene-specific probes. Black arrows indicate expression locations of *CsWOX9* in fruit epidermis. DBA, days before anthesis; Scale bars: 500 μm.

In previous studies [26,29], the expression patterns of the 11 *CsWOX* genes were examined in the cucumber variety 9930. It was found that some *CsWOXs* showed preferential expression across all tissues and organs, while others displayed obvious organ-specific expression patterns [29]. Notably, *CsWOX9* was mainly expressed in the developing ovaries of the cucumber [26]. Hence, we chose *CsWOX* genes from the cucumber variety 9930 (genome v3.0) as representatives for further study. Our findings revealed that out of the 11 detected *CsWOXs*, 5 were highly expressed in ovaries, namely *CsWOX1a*, *CsWOX4*, *CsWOX9*, *CsWOX13a*, and *CsWOX13b*. Among them, *CsWOX9* exhibited considerably higher expression level compared to the other *CsWOX* genes in the unexpanded ovaries (Figure 4A). Moreover, *CsWOX9* showed higher expression in the unexpanded ovaries than in the expanded fertilized ovaries and expanded unfertilized ovaries (Figure 4A), indicating that *CsWOX9* may play crucial roles in the morphological changes of the unexpanded ovary. In order to investigate the role of the *CsWOX9* gene in cucumber fruit, we examined its expression patterns in the epidermis and pulp using quantitative RT-PCR (qRT-PCR). The results indicated that *CsWOX9* was mainly expressed in the epidermis (Figure 4B). Consistently, an in situ hybridization assay demonstrated strong expression of *CsWOX9* in the epidermis of the cucumber ovaries (Figure 4C). Based on these findings, we hypothesize that *CsWOX9* may regulate the formation of the fruit wart phenotype.

### 2.5. Subcellular Localization of CsWOX9

**Figure 5:**
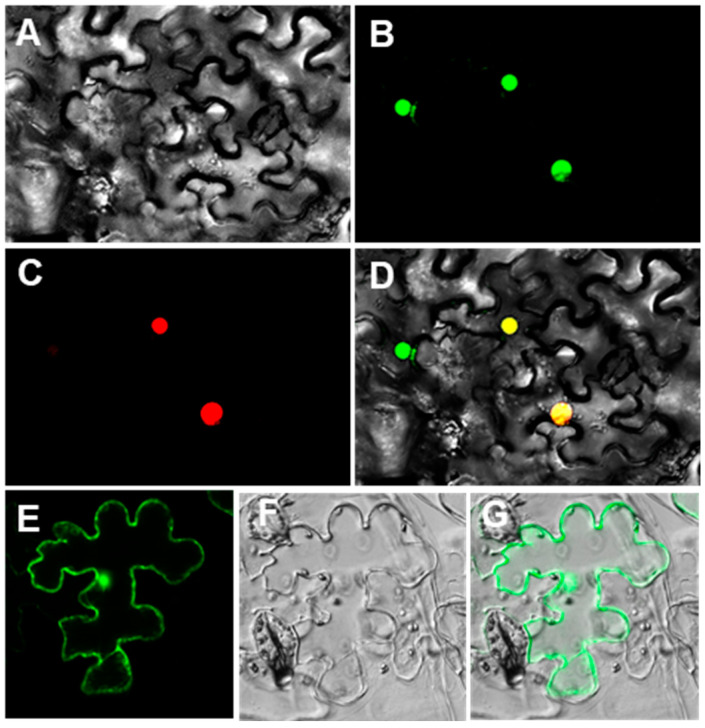
Subcellular localization of CsWOX9 protein in tobacco epidermal cells. The 35S::CsWOX9-GFP and nucleus marker NF-YA4-mCherry are co-localized in the nucleus (**A**–**D**), while the signals of 35S::GFP are detected throughout the cell (**E**,**F**). (**A**,**F**) DIC image. (**B**) CsWOX9-GFP. (**C**) Nucleus marker NF-YA4-mCherry. (**D**) Merged image of (**A**–**C**). (**E**) GFP alone. (**G**) Merged image of (**E**,**F**).

The localization of the CsWOX9 was investigated using two methods. Firstly, an online search Cell-PLoc (cellular localization of proteins) suggested that CsWOX9 might be located in the nucleus. Secondly, a tobacco transformation system was used to study the subcellular localization of CsWOX9. The 35S::CsWOX9-GFP was constructed and introduced into tobacco leaves. Fluorescence microscopy results showed that the CsWOX9-GFP fusion protein and the nucleus marker NF-YA4-mCherry were co-localized in the nucleus (Figure 5A–D). As a control, the expression of 35S::GFP was observed throughout the epidermal cell (Figure 5E–G). Consistently, Tu, a regulator of fruit Tubercule formation, was also located on the nucleus [27].

### 2.6. Tuberculate Fruit Gene Tu Directly Activates CsWOX9 by Binding to Its Promoter

**Figure 6:**
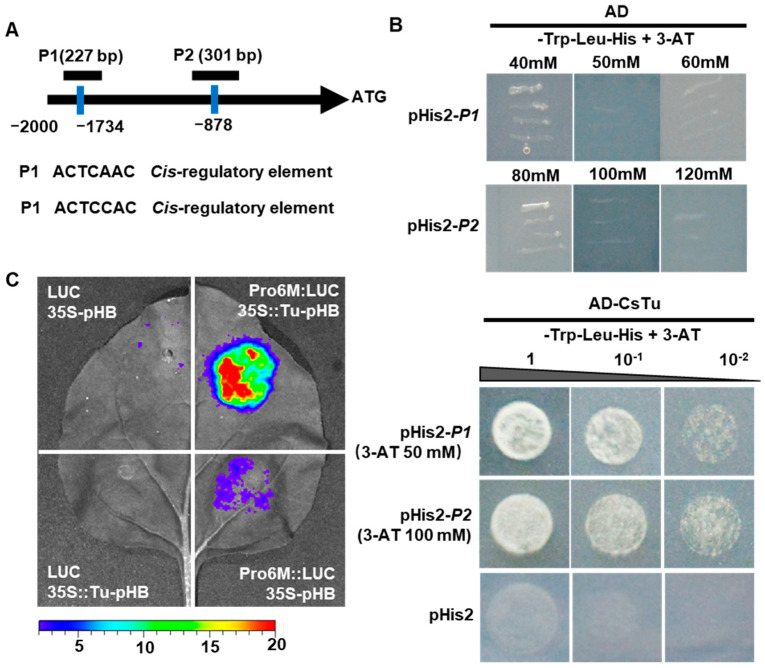
Transcriptional activation of *CsWOX9* by Tuberculate fruit gene *Tu.* (**A**) Schematic diagram of the promoter fragments of *CsWOX9* containing the binding sits of Tu used for Y1H assay. (**B**) The interaction was examined in SD medium lacking Trp, Leu, and His and containing 50/100 mM 3-AT. The empty vector pHis2 was set up as a negative control. (**C**) DLR assay showed that Tu activates the expression of *CsWOX9*.

*Cis*-elements were analyzed in the promoter of *CsWOX9*. The results revealed the identification of two cis-elements (ACTCAAC and ACTCCAC) that were bound by the C_2_H_2_ zinc finger protein (Figure 6A). The *Tu* gene, which encodes a C_2_H_2_ zinc finger protein, is required for the formation of fruit Tubercules in cucumber. This finding indicated that *Tu* may regulate *CsWOX9* transcriptionally by binding to its promoter. To test this hypothesis, two fragments, P1 and P2, containing the cis-elements ACTCAAC and ACTCCAC respectively, from the promoter of *CsWOX9*s were investigated using Y1H assays. As shown in Figure 6B, the Y187-carrying PGADT7 vectors and pHis2-*P1* or -*P2* vectors did not grow in the synthetic dextrose (SD) /-Trp/-Leu/-His medium supplemented with 50 or 100 mM 3-amino-1,2,4-triazole (3-AT), respectively. The clear positive results of the combination of AD-Tu and pHis2-*P1* or -*P2* indicated that *Tu* protein could bind to the protomer of *CsWOX9*. To further confirm this interaction, we performed a dual-luciferase reporter (DLR) assay in the leaves of tobacco (*Nicotiana benthamiana*). Compared to the vector control, the relative intensity of LUC signals significantly increased upon co-transformation of 35S::Tu with *ProCsWOX9::LUC* (Figure 6C), suggesting the activation of *CsWOX9* expression by Tu. Thus, the Y1H and DLR assays substantiated that the *Tu* gene activated the gene *CsWOX9* to regulate the formation of fruit Tubercules in cucumber.

## 3. Discussion

### 3.1. Diversification of the CsWOXs in Cucumber Pan-genome 

Studies have shown that a single reference genome is insufficient to reflect the diversity within a species [25,26,27,28]. Hence, we conducted a comprehensive analysis to identify and characterize the WOX family in 12 different cucumber varieties. Consistent with previous studies, 11 members of *CsWOX* genes were identified in cucumber 9930 genome v2.0 and v3.0 [26,29]. However, in cucumber XTMC, only nine members were identified. Furthermore, we identified 10 cucumber *WOX* genes in 10 other cucumber varieties (Table 1). These findings suggested that gene replication or deletion may have occurred during the evolutionary process, contributing to the enhancement of cucumber’s adaptability for cultivation. Interestingly, the number of *WOX* genes in cucumber is less compared to *Arabidopsis* (15) and rice (13) [1]. 

Previous study reported that the Gy14 cucumber is resistant to downy mildew, angular leaf spot and anthracnose pathogens, whereas 9930 is not. A single transition from A in 9930 to G in Gy14/WI2757 at position 323 of the *STAYGREEN* (*CsSGR*) gene coding region led to a diversity of disease resistance [37]. In this study, we observed variations in the protein length, gene structure, and conserved domains of the same *CsWOX* genes across different cucumber varieties. Additionally, the sequences of identical *CsWOX* genes with the same length in different varieties were not identical (Table 2, Figure 2, Figure 3, Figures S1 and S2). Hence, we suspect that *CsWOX* genes in different cucumber varieties have undergone distinct evolutionary changes to adapt to diverse environmental conditions. Moreover, there may be the diversity in the functions of the same *CsWOX* gene across different cucumber varieties. Further studies are needed to confirm this hypothesis. 

### 3.2. Identification of Fruit-Specific Expressed CsWOXs in Regulating Fruit Morphogenesis 

The involvement of *WOX* genes in regulating various aspects of plant growth and development has been reported [12,14,17,18,19,20]. Generally, the expression pattern of a gene can indicate its specific functions. In this study, we investigated the expression levels of all *CsWOX* genes at different developmental stages of the ovary (Figure 4A). This finding aligns with previous results that suggest the participation of the *CsWOX9* gene in physiological processes related to fruit development [26]. Additionally, our analysis using qRT-PCR and in situ hybridization assay revealed higher expression of the *CsWOX9* gene in the fruit epidermis (Figure 4B,C). Consequently, we identified the *CsWOX9* gene as an important regulator controlling the morphogenesis of cucumber ovary epidermis.

**Figure 7:**
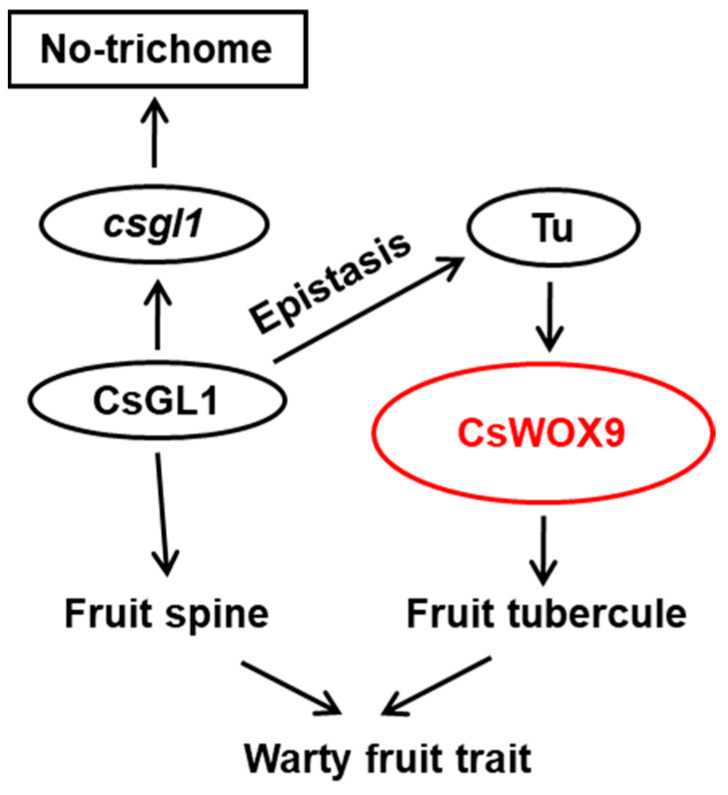
A proposed genetic network of *CsWOX9* involved in regulating the formation of the fruit warts in cucumber. *CsGL1* was required for the further differentiation of cucumber trichomes, and the *csgl1* mutant exhibited glabrous ovaries/fruits. Furthermore, *CsGL1* was essential for *Tu* expression, as the absence of *CsGL1* resulted in the lack of *Tu* expression and consequently the absence of Tubercules. Thus, *csgl1* was epistatic to the *Tu* gene. The *Tu* was upregulated before Tubercules’ formation, then activating the expression of *CsWOX9* gene transcriptionally, induced the formation of Tubercules.

In this study, the localization of CsWOX9 was investigated. Consistent with previous study of Tu [27], CsWOX9 was found to be located in the nucleus (Figure 5A–D). Additionally, the promoter of *CsWOX9* was observed to contain two potential binding sites of ACTCA/CAC, which are known to be key binding sites for C_2_H_2_ zinc finger TFs in plants [38] (Figure 6A). The gene *Tu* encodes a C_2_H_2_ zinc finger protein and is known to regulate fruit Tubercule formation. To further understand the transcriptional regulatory mechanism, we performed Y1H and dual-LUC transaction assays. The results demonstrated that Tu activates the expression of *CsWOX9* by binding to its promoter region (Figure 6B,C). Previous studies have shown that *CsGL1* is necessary for fruit spine formation and the expression of *Tu* [27,39]. Based on these findings, we propose a possible model in which *CsWOX9* is transcriptionally activated by Tu, leading to the regulation of fruit wart formation (Figure 7).

It was reported that overexpression of *CsWOX9*, a homolog of *AtWOX9*, affected the plant architecture and fruit length in *Arabidopsis* [26]. Tiller growth in rice was dependent on the presence of *DWARF TILLER1* (*DWT1*), which is the homolog of *AtWOX8/9* [40]. Additionally, both *MtWOX9* and *NsWOX9* are orthologs of *AtWOX9*, and they are necessary for the outgrowth of leaf blade in *Medicago truncatula* and *Nicotiana sylvestris*, respectively [41]. *EVG*, a homolog of *AtWOX9* in petunia, is essential for the appropriate branching of the inflorescence apex and plays a role in determining the identity of the floral meristem [42]. The findings indicated that WOX9 TFs, which exhibited structural conservation, played various roles in regulating the development and growth of plants across different species. Through this research, we discovered that *CsWOX9* likely plays a role in controlling the formation of fruit warts, suggesting its distinct functionality when compared to homologs in other species.

## 4. Materials and Methods

### 4.1. Identification and Phylogenetic Analysis of CsWOX Genes

To identify *CsWOX* genes in the 12 cucumber genomes (https://www.ncbi.nlm.nih.gov/, accessed on 11 August 2023), the full-length sequences of 15 AtWOX members obtained from the *Arabidopsis* genome database (https://www.arabidopsis.org, accessed on 11 August 2023) were used for the hidden Markov model (HMM) construction. These varieties included East-Asian lines 9930, XTMC, and Cu2; Eurasian lines Cuc37, Gy14, and 9110gt; Xishuangbanna line Cuc80; and Indian lines Cuc64, W4, W8, Hx14, and Hx117 (Table S1). Subsequently, the HMM file was used for the identification of *WOX* genes from 12 cucumber genomic databases by HMMER 3.0. The sequences of the candidate members were further analyzed using Pfam (http://pfam.xfam.org, accessed on 12 August 2023) and SMART (http://smart.embl-heidelberg.de, accessed on 12 August 2023) to confirm the existence of the HD.

Alignments of the amino acids sequences of WOX family members from *Arabidopsis* and cucumber were performed using ClustalW in MEGA 7.0 and used to construct the phylogenetic tree with the neighbor-joining (NJ) method. The tree was visualized and optimized by Evolview (http://www.evolgenius.info/evolview, accessed on 5 October 2023).

### 4.2. Bioinformatics Analysis of CsWOX Genes

CsWOX TFs, which vary in protein length in at least three different cucumber varieties, were selected for further analyzed. The amino acids sequences of these selected *WOX* genes from 12 cucumber varieties were obtained based on the pan-genome. The motifs were analyzed using the MEME online program (https://meme-suite.org/meme/tools/meme, accessed on 17 August 2023). The amino acids sequences were aligned using ClustalW in MEGA 7.0 and this alignment was used to generate the phylogenetic tree with the maximum likelihood method. Combination images of phylogenetic clustering, conserved protein motifs and gene structure of *CsWOX* genes were visualized and optimized using the TBtools software (v2.012; Guangzhou, China).

### 4.3. Transcriptome Analysis of WOX Genes in Cucumber Ovaries

The expression profiles of the *CsWOX* genes were analyzed based on published RNA-seq data (SRA046916) [43]. FPKM values were recalculated through clean tags remapping to the cucumber 9930 genome sequence (http://cucurbitgenomics.org/, v3.0, accessed on 25 August 2023) by Biomarker Technologies (Beijing, China). These analyses were performed on cucumber ovaries on different development stages, including unexpanded ovaries, expanded unfertilized ovaries, and expanded fertilized ovaries. There was only one biological replication for each tissue sample. The expression patterns of the *CsWOX* genes were shown on a heat map using the TBtools software (v2.012; Guangzhou, China).

### 4.4. Expression Analysis of CsWOX9 by qRT-PCR

Total RNA was extracted using Total RNA Isolation System (Huayueyang, Beijing, China) and used to synthesize cDNA with the EasyScript^®^ One-Step gDNA Removal and cDNA Synthesis SuperMix (TransGen Biotech, Beijing, China). qRT-PCR was performed using the 2X M5 HiPer Realtime PCR Super mix (Mei5 Biotechnology, Beijing, China) with an Applied Biosystems 7500 real-time PCR system. The cucumber *UBI* (*CsaV3_5G031430*) gene was used as internal controls. Three biological replicates were used for expression analysis. Primers were listed in Table S2.

### 4.5. In Situ Hybridization

The unexpanded ovaries (at 7 DBA) were fixed in 3.7% FAA followed with both graded EtOH treatments (50%, 70%, 85%, 95% and 100%, each EtOH concentration for 90 min at 4 °C) and then xylene treatment (50% EtOH/50% xylene for one time, 100% xylene for two times, all treatments at room temperature), embedded with par-aplast after paraplast infiltration for 3 days at 60 °C, sectioned with a microtomes, and hybridized with digoxigenin-labeled sense and antisense gene-specific probe as previous described [44]. The primers with T7 and SP6 RNA polymerase-binding sites were listed in Table S2.

### 4.6. Subcellular Localization of CsWOX9

The coding region of *CsWOX9* without the stop codon was cloned and fused upstream of the GFP sequence between the *Xba*I and *Spe*I sites of the pCAMBIA1300 vector, which was modified for this study, to generate 35S::CsWOX9-GFP. The constructs of 35S::CsWOX9-GFP and 35S::GFP were introduced into *Agrobacterium* strain EHA105 by electroporation, respectively. Agrobacterium cultures carrying 35S:CsWOX9-GFP or 35S::GFP constructs were then infiltrated into tobacco leaves using a needleless syringe. The fluorescence signals were measured at 3 days post infiltration using a Laser Scanning Confocal Microscope 880 (Carl Zeiss AG, Jena, Germany). Primers are listed in Table S2.

### 4.7. Y1H Assay

The coding sequence of *Tu* was cloned into the pGADT7 prey vector. Two fragments of *CsWOX9* promoter (P1 and P2) containing the C_2_H_2_ zinc finger TFs binding sites ACTCA/CAC were shown as the bait sequences. Bait sequences were cloned into the pHis2 vector by using the primers listed in Table S2. The indicated plasmid pairs were transformed into the yeast strain Y187 according to the manufacturer’s instructions (Clontech, Mountain View, CA, USA). The transformed yeast cells carrying an empty pGADT7 vector were used as negative controls. Yeast cells were grown on SD medium lacking Trp and Leu. The transformants were subsequently transferred to SD medium without Trp, Leu, and His, supplemented with 50/100 mM 3-AT. They were cultured for 3–4 days to monitor yeast growth. The experiments were repeated at least three times.

### 4.8. Dual-LUC Transaction Assay

For study of the binding activity of Tu to the promoter of *CsWOX9*, the coding sequence of *Tu* was cloned into the pHB vector at the *Hind*III and *BamH*I sites. Simultaneously, the promoter of *CsWOX9* (2000 bp) was cloned into the pGreenII 0800-LUC double-reporter vector at the *Hind*III and *BamH*I sites [45]. The generated constructs were then transformed into *Agrobacterium* strain EHA105 and then co-infiltrated into tobacco leaves as above. The firefly LUC activities were measured using vivo imaging system Lumina (Xenogen, Hopkinton, MA, USA).

## 5. Conclusions

Based on the pan-genome, we identified 11 *CsWOX* genes. We also observed variations in the protein length and sequence, gene structure, and conserved domains of the same *CsWOX* genes across different cucumber varieties. Therefore, we hypothesize that *CsWOX* genes in different cucumber varieties have undergone distinct evolutionary changes to adapt to diverse environmental conditions. *CsWOX9* was found to be a fruit-specific expressed gene, particularly in the fruit epidermis. Additionally, the promoter of *CsWOX9* interacted with Tu, a requirement for fruit Tubercule formation in cucumber, as confirmed by the Y1H experiment and DLR assay. In this study, a draft model was constructed to demonstrate that *CsWOX9* is transcriptionally activated by Tu, resulting in the regulation of fruit wart formation.

## Figures and Tables

**Table 1:** Identification of *WOX* genes in different cucumber varieties.

Gene Name	Gene ID ^1^												
	9930-V3 *	9930-V2 *	XTMC	Cu2	Cuc37	Cuc64	Cuc80	W4	W8	Hx14	Hx117	Gy14	9910gt
*CsWOX1a*	*1G007120*	*1G042780*	*1G007180*	*1G007360*	*1G007230*	*1G007200*	*1G007190*	*1G007200*	*1G007230*	*1G013410*	*1G010430*	*1G012440*	*1G007480*
*CsWOX1b*	*1G004310*	*1G025040*	*1G004260*	*1G004440*	*1G004350*	*1G004310*	*1G004310*	*1G004370*	*1G004340*	*1G009460*	*1G007470*	*1G008510*	*1G004470*
*CsWOX2*	*1G034050*	*1G505930*	-	-	-	-	-	-	-	-	-	-	-
*CsWOX3*	*6G021960*	*6G301060*	*6G032580*	*6G023220*	*6G018920*	*6G021510*	*6G052960*	*6G019330*	*6G022530*	*6G032230*	*6G029450*	*6G030400*	*6G023370*
*CsWOX4*	*2G026510*	*2G356610*	*2G028100*	*2G027900*	*2G097530*	*2G067930*	*2G100850*	*2G032030*	*2G040170*	*2G036130*	*2G036990*	*2G034990*	*2G028340*
*CsWOX7*	*6G001670*	*6G010010*	*6G001650*	*6G001610*	*6G001650*	*6G001660*	*6G003640*	*6G001620*	*6G001620*	*6G002610*	*6G001650*	*6G004680*	*6G001650*
*CsWOX9*	*6G050540*	*6G518270*	*6G062220*	*6G046400*	*6G046950*	*6G045020*	*6G100570*	*6G043880*	*6G044120*	*6G063500*	*6G055780*	*6G056710*	*6G047130*
*CsWOX11*	*3G039320*	*3G812740*	*3G059960*	*3G046380*	*3G055190*	*3G060870*	*3G051480*	*3G046100*	*3G044550*	*3G064800*	*3G065520*	*3G060130*	*3G046900*
*CsWOX13a*	*3G000330*	*3G002330*	-	*3G000300*	*3G000320*	*3G000320*	*3G000280*	*3G000310*	*3G000300*	*3G000320*	*3G000310*	*3G000330*	*3G000310*
*CsWOX13b*	*4G037370*	*4G663700*	*4G049750*	*4G040360*	*4G101470*	*4G038090*	*4G101290*	*4G033260*	*4G039360*	*4G048450*	*4G045640*	*4G047420*	*4G042180*
*CsWUS*	*6G047050*	*6G505860*	*6G058810*	*6G042980*	*6G043520*	*6G041670*	*6G097310*	*6G040480*	*6G040750*	*6G059150*	*6G052360*	*6G052360*	*6G043720*

^1^ The gene IDs in the table do not include abbreviations that could represent different cucumber varieties. * V3, genome v3.0; V2, genome v2.0. - indicates the WOX gene was not identified in this cucumber cultivar.

**Table 2:** The predicted lengths of WOX proteins (amino acid residues) in different cucumber cultivars.

Protein Name	9930_V3 *	9930_V2 *	XTMC	Cu2	Cuc37	Cuc64	Cuc80	W4	W8	Hx14	Hx117	Gy14	9910gt
CsWOX1a	390	387	390	339	390	389	390	390	388	390	390	390	339
CsWOX1b	334	334	334	334	334	333	333	334	334	334	334	334	334
CsWOX2	239	239	-	-	-	-	-	-	-	-	-	-	-
CsWOX3	193	193	193	193	193	187	193	193	198	193	187	193	193
CsWOX4	227	227	227	227	227	228	226	227	224	227	226	227	227
CsWOX7	196	130	116	196	196	196	196	196	196	196	180	168	196
CsWOX9	350	376	376	376	376	376	376	376	376	376	376	376	168
CsWOX11	257	257	259	357	276	319	257	370	417	301	396	257	257
CsWOX13a	201	282	-	282	282	282	282	282	282	282	282	282	282
CsWOX13b	314	269	269	269	317	269	317	317	317	317	317	269	317
CsWUS	233	304	233	233	233	233	233	230	233	233	233	233	233

* V3, genome v3.0; V2, genome v2.0. - indicates the *WOX* gene was not identified in this cucumber cultivar.

## Data Availability

Data is contained within the article and supplementary material.

## Supplementary Materials

The following supporting information can be downloaded at: https://www.mdpi.com/article/10.3390/ijms242417568/s1.

## Abbreviations

Y1H	yeast one-hybrid assay
DLR	dual-luciferase reporter
WOX	WUSCHEL-related homeobox
HD	homeodomain
TFs	transcription factors
WUS	WUSCHEL
RAMs	root apical meristems
EVG	EVERGREEN
PFS2	PRETTY FEW SEEDS2
a.a.	amino acids
qRT-PCR	quantitative RT-PCR
3-AT	3-amino-1,2,4-triazole
SGR	STAYGREEN
DWT1	DWARF TILLER1
SD	synthetic dextrose
DBA	days before anthesis
