# Supplementary material for: Pan-genome Analysis of WOX Gene Family and Function Exploration of CsWOX9 in Cucumber
Source: Int J Mol Sci. 2023 Dec 17;24(24):17568. doi: 10.3390/ijms242417568 (PMC10743939; doi:10.3390/ijms242417568)
Supplement: Supplementary file 1 [file ijms-24-17568-s001.zip › Supplementary Figures.pdf]

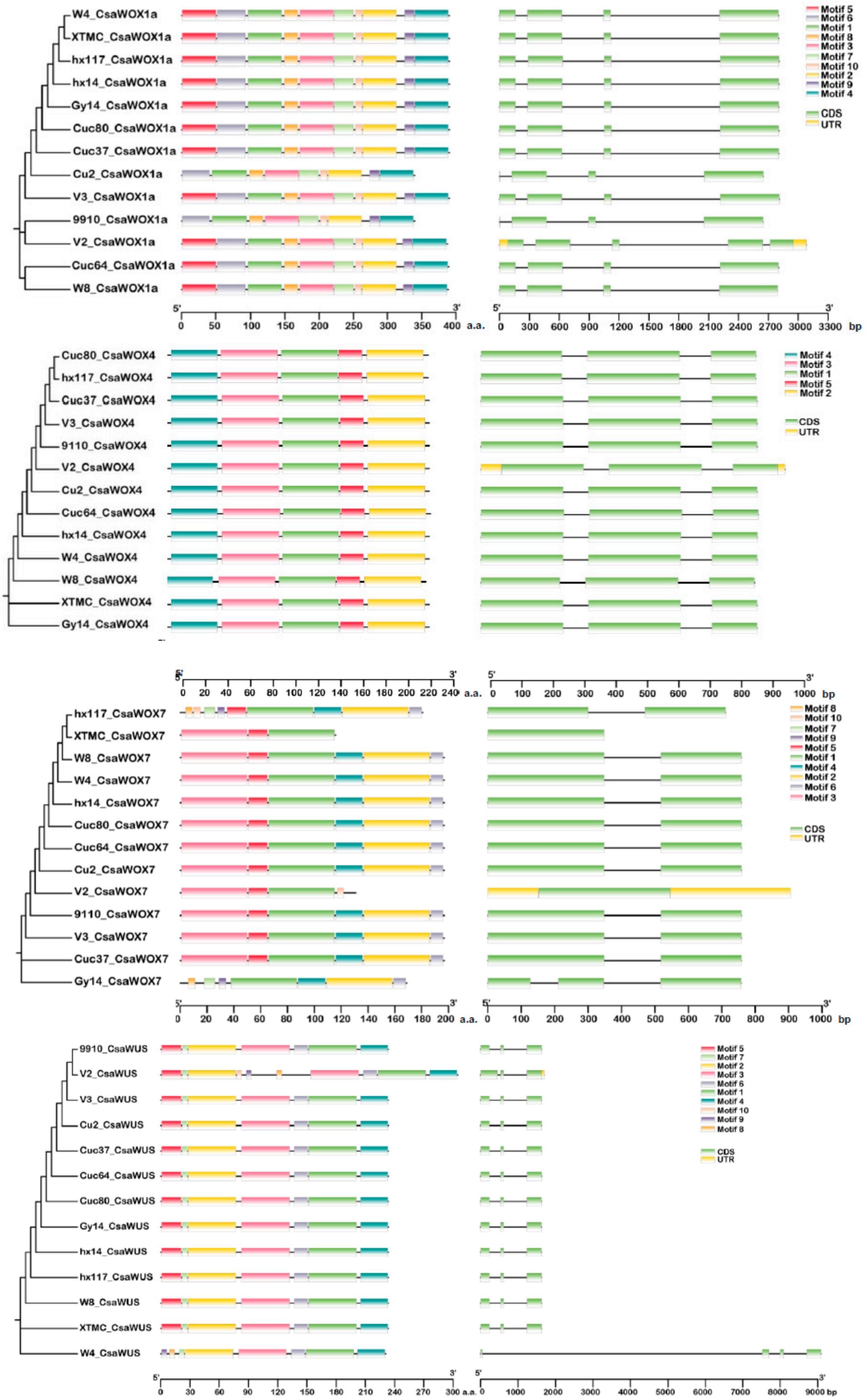
W4_CsaWOX1a
XTMC_CsaWOX1a
hx117_CsaWOX1a
hx14_CsaWOX1a
Gy14_CsaWOX1a
Cuc80_CsaWOX1a
Cuc37_CsaWOX1a
Cu2_CsaWOX1a
V3_CsaWOX1a
9910_CsaWOX1a
V2_CsaWOX1a
Cuc64_CsaWOX1a
W8_CsaWOX1a
Motif 5
Motif 6
Motif 1
Motif 8
Motif 3
Motif 7
Motif 10
Motif 2
Motif 9
Motif 4
CDS
UTR
5'
3'
0 50 100 150 200 250 300 350 400 a.a.
0 300 600 900 1200 1500 1800 2100 2400 2700 3000 3300 bp
Cuc80_CsaWOX4
hx117_CsaWOX4
Cuc37_CsaWOX4
V3_CsaWOX4
9110_CsaWOX4
V2_CsaWOX4
Cu2_CsaWOX4
Cuc64_CsaWOX4
hx14_CsaWOX4
W4_CsaWOX4
W8_CsaWOX4
XTMC_CsaWOX4
Gy14_CsaWOX4
Motif 4
Motif 3
Motif 1
Motif 5
Motif 2
CDS
UTR
0 20 40 60 80 100 120 140 160 180 200 220 240 a.a.
0 100 200 300 400 500 600 700 800 900 1000 bp
hx117_CsaWOX7
XTMC_CsaWOX7
W8_CsaWOX7
W4_CsaWOX7
hx14_CsaWOX7
Cuc80_CsaWOX7
Cuc64_CsaWOX7
Cu2_CsaWOX7
V2_CsaWOX7
9110_CsaWOX7
V3_CsaWOX7
Cuc37_CsaWOX7
Gy14_CsaWOX7
Motif 8
Motif 10
Motif 7
Motif 9
Motif 5
Motif 1
Motif 4
Motif 2
Motif 6
Motif 3
CDS
UTR
0 20 40 60 80 100 120 140 160 180 200 a.a.
0 100 200 300 400 500 600 700 800 900 1000 bp
9910_CsaWUS
V2_CsaWUS
V3_CsaWUS
Cu2_CsaWUS
Cuc37_CsaWUS
Cuc64_CsaWUS
Cuc80_CsaWUS
Gy14_CsaWUS
hx14_CsaWUS
hx117_CsaWUS
W8_CsaWUS
XTMC_CsaWUS
W4_CsaWUS
Motif 5
Motif 7
Motif 2
Motif 3
Motif 6
Motif 1
Motif 4
Motif 10
Motif 9
Motif 8
CDS
UTR
0 30 60 90 120 150 180 210 240 270 300 a.a.
0 1000 2000 3000 4000 5000 6000 7000 8000 9000 bp

1

Figure S1. Phylogenetic relationships, conserved motifs and gene structure of WUS clade genes.

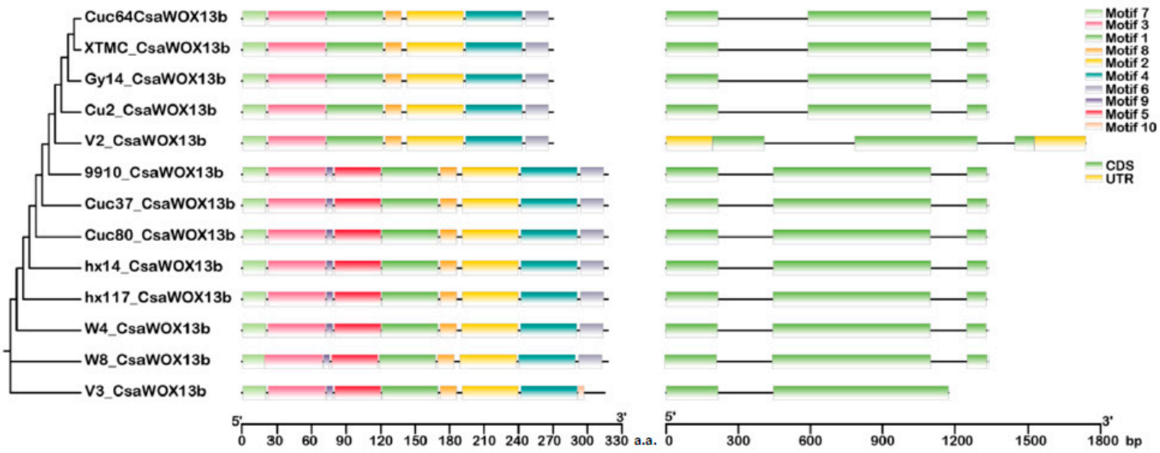

Figure S2. Phylogenetic relationships, conserved motifs and gene structure of Ancient clade gene *CsWOX13B*.
