# Supplementary material for: Pan-genome Analysis of WOX Gene Family and Function Exploration of CsWOX9 in Cucumber
Source: Int J Mol Sci. 2023 Dec 17;24(24):17568. doi: 10.3390/ijms242417568 (PMC10743939; doi:10.3390/ijms242417568)
Supplement: Supplementary file 1 [file ijms-24-17568-s001.zip › Supplementary Tables.pdf]

Table S1 The origins of different cucumber lines

**Accession**	**Group**	**Fruit morphology**	**Accessio n**	**Group**	**Fruit morphology**
9930	East-Asian	Dense warts	9110gt	Eurasian	Dense warts
XTMC	East-Asian	Dense warts	Cuc64	Indian	Lack tubercules
Cu2	East-Asian	Dense warts	W4	Indian	Sparse warts
Cuc80	Xishuangbanna	Lack tubercules	W8	Indian	Sparse warts
Cuc37	Eurasian	Lack tubercules	Hx14	Indian	Lack tubercules
Gy14	Eurasian	Sparse warts	Hx117	Indian	Lack tubercules

Table S2. Primer information used in this study

Primers for qRT-PCR (5'-3')	
UBI-F	CACCAAGCCCAAGAAGATC
UBI-R	TAAACCTAATCACCACCAGC
CsWOX9-F	ATTTCCACGCCTCTCACCAC
CsWOX9-R	GGAGAGTGAGACCCCAGTCA
Primers for *In Situ* Hybridization (5'-3')	
CsWOX9-SP6	GATTTAGGTGACACTATAGAATGCTTCCCAAACCACTACAACA ACC
CsWOX9-T7	TGTAATACGACTCACTATAGGGAGAGGCGTGGAAATAGTGGA
Primers for gene cloning (5'-3')	
Tu-F	ATGGCAGCTCTAGAAAACCA
Tu-R	TGGGGCGGCCGGAGCGAG
CsWOX9-F	ATGGCTTCCTCTAACAGACACTGGC
CsWOX9-R	TATCAGATAATAGTAAGAACCATG
Primers for promoter cloning (5'-3')	
ProCsWOX9-F	TCTTTTTGTTATAATTTGTGAAA
ProCsWOX9-R	GAAAATATATGATATGCTGCTGCCCT
ProP1-F	5TGCCTTCAACTAAAAGCCTTCA
ProP1-R	GGGAAAGAGGGGGTTTGGTT
ProP2-F	TGAAATGGCCTAGAGAAATGAAATGA
ProP1-R	GGGGTGAGAAGAGGTAGAGAC
